# Household secondhand smoke exposure of elementary schoolchildren in Southern Taiwan and factors associated with their confidence in avoiding exposure: a cross-sectional study

**DOI:** 10.1186/1471-2458-12-40

**Published:** 2012-01-17

**Authors:** Hsiao-Ling Huang, Yea-Yin Yen, Pi-Li Lin, Chin-Hsuan Chiu, Chih-Cheng Hsu, Ted Chen, Chih-Yang Hu, Ya-Ying Lin, Chien-Hung Lee, Fu-Li Chen

**Affiliations:** 1 Department of Oral Hygiene, College of Dental Medicine, Kaohsiung Medical University. 100 Shih-Chuan 1st Road, Kaohsiung City 80708, Taiwan; 2 Department of Nursing, Meiho University. 23, Pingguang Rd., Neipu Town, Pintung County 91202, Taiwan; 3 Division of Preventive Medicine and Health Services Research, Institute of Population Health Sciences, National Health Research Institutes. No. 35, Keyan Road, Zhunan Town, Miaoli County 350, Taiwan; 4 Department of Global Community Health and Behavioral Sciences, School of Public Health and Tropical Medicine, Tulane University, New Orleans, LA 70112, USA; 5 School of Public Health, Health Sciences Center, Louisiana State University, New Orleans, LA 70112, USA; 6 Department of Physical Therapy, Shu Zen College of Medicine and Management. No. 452, Huanqiu Rd., Luzhu District, Kaohsiung City 82144, Taiwan; 7 Department of Public Health, College of Health Science, Kaohsiung Medical University. 100 Shih-Chuan 1st Road, Kaohsiung City 80708, Taiwan; 8 Department of Public Health, Fu-Jen Catholic University. 510 Jhongjheng Rd., Sinjhuang District, New Taipei City 24205, Taiwan

**Keywords:** Children, Secondhand smoke

## Abstract

**Background:**

Exposure to household Secondhand Smoke (SHS) poses a major health threat to children after an indoor smoking ban was imposed in Taiwan. This study aimed to assess the household SHS exposure in elementary school children in southern Taiwan and the factors associated with their avoidance of SHS exposure before and after the implementation of Taiwan's new Tobacco Hazards Prevention Act in 2009.

**Methods:**

In this cross-sectional school-based study, data on household SHS exposure, avoidance of SHS and related variables was obtained from the 2008 and 2009 Control of School-aged Children Smoking Study Survey. A random sample of 52 elementary schools was included. A total of 4450 3-6 graders (aged 8-13) completed the questionnaire. Regression models analyzed factors of children's self-confidence to avoid household SHS exposure.

**Results:**

Over 50% of children were found to have lived with a family member who smoked in front of them after the new law enacted, and 35% of them were exposed to household SHS more than 4 days a week. Having a positive attitude toward smoking (β = -0.05 to -0.06) and high household SHS exposure (β = -0.34 to -0.47) were significantly associated with a lower avoidance of SHS exposure. Comparing to girls, boys had lower scores in their knowledge of tobacco hazards; and this factor was significantly related to their SHS avoidance (β = 0.13-0.14).

**Conclusions:**

The intervention program should enhance school children do actively avoid exposure to SHS in home settings, and more importantly, provide tobacco hazard knowledge to male students to avoid exposure to household SHS for themselves. The results also provide further evidence that Tobacco Hazards Prevention Act should perhaps be extended to the family environment in order to protect children from the hazards of household SHS exposure.

## Background

It is universally known that cigarette smoking is harmful to health, and much evidence shows that exposure to Secondhand Smoke (SHS) also poses great harm to the health [1,2]. Research has shown that SHS poses a greater health risk to children than to adults [3]. Children who are exposed to secondhand smoke are at an increased risk for sudden infant death syndrome, lower respiratory infections, middle ear disease, more severe asthma, respiratory symptoms, and slowed lung growth [4,5].

Children are more heavily exposed to secondhand smoke at home than adults. Since the Taiwanese smoking population was recently estimated at 3.61 million, 35.4% of the male and 4.2% of the female population smoked [6], much of the general public is quite vulnerable to SHS exposure. According to the survey on smoking behavior conducted by the Taiwanese Bureau of Health Promotion, 46.8% of junior high school students self-reported having SHS exposure at home over the past 7 days [7]. The aforementioned findings indicate that younger non-smokers are more vulnerable to SHS exposure at home. Another recent study in Taiwan [8,9] found that 67.5% of the children have family members smoking at home and that 53.9% of them smoked in front of these elementary students.

The Tobacco Hazards Prevention Act, first enacted in Taiwan in 1997, aims to prevent tobacco hazards such as child exposure to SHS, to prohibit children and adolescents from smoking and has established regulations with respect to where tobacco use is restricted. The amendments to the Act in 2009 stipulated that tobacco use is strictly prohibited in indoor public spaces and workplaces. These amendments, also further aimed to reduce SHS in children by expanding the prohibition of tobacco use in any indoor area with the presence of pregnant women and/or children under the age of three. While this act protects children and other non-smokers from SHS exposure in public spaces, and pregnant women and children under the age of 3 from SHS exposure in-doors it does not protect all children from SHS exposure within the home environment.

One's confidence in regard to avoidance behavior of SHS plays a critical role in its exposure to SHS. Self-confidence and SHS avoidance behavior in the youth were found inversely associated with the number of family members who smoke at home after Taiwan's regulatory partial smoking bans in some public and private places [10]. These results indicate that the youth was extremely confident of their SHS avoidance behavior in the school setting, whereas was not at all confident of their avoidance of SHS in the home setting. Most studies have focused on the status quo of SHS exposure or avoidance behaviors on adolescents and adults [10-12]. As a result, there is a research need to better understand elementary children's SHS exposure at home and their avoidance behavior of SHS exposure. Hence, this study aimed to assess the degree of household SHS exposure and factors associated with confidence level in avoiding SHS exposure in elementary school children in southern Taiwan, both before and after the implementation of the newly amended Act.

## Methods

Data on household SHS exposure, children's perceived actual avoidance of SHS exposure and on related variables for elementary children were obtained from the Control of School-aged Children Smoking Study (CSS study) of 2008-2009. This CSS study was explicitly indicated elsewhere [9].

### Sampling design and participants

Multistage cluster sampling was applied, where the sampling unit is the school for this study. Elementary schools were randomly selected based on stratification of three geographic areas (urban, rural, and mountainous areas). The mountainous school sampled has only approximately 30 students from 3rd to 6th grade per school, as compared to about 400 students in a typical rural or urban school. A total of 10 mountain schools, 8 rural schools and 8 urban schools were randomly selected from a composite list of elementary schools provided by the Education Bureau of Pin-Tung City and County, Kaohsiung City and County in southern Taiwan in 2008 and 2009, respectively. None of the randomly selected schools declined to participate in the study. Four classes were randomly selected for each school; one class each from each school year of 3-6 grades (aged 8-13). For the purpose of this study, we used data of 26 elementary schools from Ping-Tung City and County in 2008 before the new act (wave 1) and then an additional 26 elementary schools from Kaohsiung City and County after the implementation of the newly amended Act in 2009 (wave 2). In sum, 2,492 questionnaires were obtained in the wave 1 and 2,337 questionnaires were obtained in the wave 2. The response rate was 98.1% and 98.9%, respectively.

The surveys were administered by well-trained members of the research team in the classroom for the spring semesters in 2008 and 2009. The purpose and instructional procedure of the survey were explained to each student prior to taking the questionnaire. A parental informed consent was obtained from all of the participants. This consent procedure consisted of sending an introduction letter home with the child describing the study and requesting parents or legal guardians to submit a signed consent form if they wish their child to participate in the study. The parents or legal guardians were also asked to fill out a questionnaire designed for parents and return it in a sealed envelope to the researcher within 1 week. No personal identifier was asked for both student and parent questionnaires.

### Questionnaire

The survey questionnaire, which contains 103 items, was adopted and modified from an established and validated questionnaire from the Global Youth Tobacco Survey (GYTS) and the Child and Adolescent Behaviours in Long-Term Evolution (CABLE) study [13,14]. This survey instrument was also proved effective in the survey of smoking behaviours for elementary students in Taoyuan, Taiwan [15-17]. The structured self-administered questionnaire was also reviewed by a panel of experts, school teachers and parents to assess its face and content validity. To ensure that the content was age-appropriate, a pilot-test was conducted on a convenient sample of third graders in Pin-Tung County.

### Constructive variables

#### Household secondhand smoke exposure

The frequency of the students' household SHS exposure was assessed by the question: "Over the past week, how many days did people smoke in front of you while you were at home?" Response choices included "None," "1-3 days," "4-6 days," and "everyday". The household SHS exposure was then classified into "none," "1-3 days/week," and "4+ days/week".

#### Outcome variable

Two questions were used to measure students' confidence level of perceived actual avoidance of SHS exposure at home: "Are you confident that you do not inhale Secondhand Smoke while being with relatives or elders at home?" and "Are you confident that you do not inhale Secondhand Smoke while being with visitors at home?" The response choices were on a 4-point scale and ranged from "Definitely" to "Not at all". The total score ranged from 2 to 8, with a high score indicated that a high level of household SHS avoidance confidence and that they were more confident that they did not inhale Secondhand Smoke while being with a smoker(s) at home.

#### Independent variable

The independent variables include socio-demographic factors, Secondhand Smoke factors and socio-psychological factors. Socio-demographic factors consist of gender (male/female), grade, ethnicity (non-aboriginal/aboriginal), school geography (urban/rural/mountain), monthly household income (< $40,000/$40,000-$80,000/> $80,000 in Taiwanese Dollars), and parent's highest educational level (less than high school/high school/college and up).

The variable 'smoking behavior' was assessed with the question "Have you *ever *smoked cigarettes (even just one puff)?" The possible responses were (1) I do not smoke; (2) less than one cigarette; (3) less than 10 cigarettes; (4) more than 10 cigarettes. Never-smokers were defined as those students who reported that they had never smoked a cigarette, not even a puff. Those who reported that they had smoked even just a puff or more were required to answer the following question "Have you smoked cigarettes over the past month?" Students who answered "No" to this question were defined as former smokers, while those who answered "Occasionally" or "Everyday" were defined as current smokers. Family smoking levels were assessed by asking students two items: (a) Is there anyone living in the same household with you that smokes? ('Yes'/'No') and (b) How often do they smoke in front of you? ('Always'/'Sometimes'/'Never').

Ten interrogative statements were used to evaluate students' knowledge level of tobacco hazards. An example of the statement include: "As long as we do not inhale second-hand smoke, smoking is not dangerous". Response choices to these statements were "*True"*, "*False"*, and "*I do not know"*. One point was assigned for each correct answer, and no point was assigned for incorrect answer or for the answer "*I do not know*". The total score range was from 0 to 10 points. The reliability coefficient for the knowledge scale was 0.76, indicating that internal consistency was adequate. A set of 12 statements were used to evaluate students' attitude toward smoking. The variable corresponding to students' attitude toward smoking was consolidated by assigning a higher score to responses that were favorable to smoking, and a lower score to those that were unfavorable. Examples of the statements include: "Smoking makes people happy", "No one is allowed to smoke in schools". A 4-point scale ranging from 1 (strongly disagree) to 4 (strongly agree) was employed. Reverse scoring was applied to negative statements. The sum of theses scores then represent a quantitative expression of students' attitude toward smoking. The students with the most favorable attitudes toward smoking had thus the highest sum. The reliability coefficient of the attitude scale was 0.73, indicating that the items were internally consistent. An overview of the survey items of independent variables is illustrated in Additional file 1: Table S1 and Additional file 2: Table S2.

#### Research ethic approval

The study was approved by the Institutional Review Board of Kaohsiung Medical University Hospital. Written informed consent was obtained from the parents or legal guardians of participants before taking surveys.

#### Statistical analysis

SPSS Version 12.0 was employed to conduct our statistical analysis. The analysis was performed in order to explore the influence of the socio-demographic factors, socio-psychological factors and the SHS factors on household SHS avoidance confidence on the basis of the survey results. The chi-square test and independent *t*-test were applied to nominal or ordinal variables of the socio-demographic factors, to the socio-psychological factors, as well as to the SHS factors. This study then probed into whether the variance of each relevant factor had any significant association to SHS avoidance confidence at home. Both univariate and multivariate regression models were employed in order to assess the unadjusted and adjusted association, respectively, for male and female students in the springs of both 2008 and 2009. Finally in order to analyze the change in outcome variable predicted or attributable by the independent variables, only the factors that were found to be significant in the univariate regression were included in the multiple regression models. β coefficients and its 95% confidence interval were reported in multiple regression models about confidence level as to the avoidance of household SHS exposure associated with its potential factors. The significance level then was determined based on *P *values less than 0.05.

## Results

### Study participants

The final valid sample for the study as shown in Table 1 included the 2,292 students (51.5%) in wave 1 survey in 2008 and the 2,158 students (48.5%) in wave 2 survey in 2009. Among this sample, the wave 2 sample had a higher aboriginal rate than that of the wave 1 sample (24.3% vs. 17.0%). The rest of the demographic characteristics were distributed in a relatively similar manner between wave 1 and 2.

**Table 1:** The demographic profile based on the questionnaire respondents and presented as proportions within the respective survey groups. (n = 4,450)

	N	%	Wave 1^a^	Wave 2^b^	*P *value
Regional					
Pin-Tung	2292	51.5	-----	-----	
Kaohsiung	2158	48.5	-----	-----	
Gender					
Male	2237	50.3	51.7	48.7	0.046
Female	2213	49.7	48.3	51.3	
Grade					
3	1057	23.8	23.9	23.6	0.925
4	1099	24.7	25.0	24.3	
5	1120	25.2	25.0	25.3	
6	1174	26.4	26.1	26.7	
Ethnicity					
Non-aboriginal	3000	67.4	61.4	73.8	0.025
Aboriginal	915	20.6	17.0	24.3	
School Geography					
Urban	1923	43.2	42.1	42.5	0.358
Rural	1662	37.3	33.2	34.6	
Mountain	865	19.4	24.7	23.0	
Monthly household income					
High (> $80,000)	557	17.8	19.3	16.4	0.039
Middle ($40,000-$80,000)	1081	34.6	33.7	35.5	
Low (< $40,000)	1483	47.5	47.0	48.1	
Mother's educational attainment					
College or higher	287	8.3	8.9	7.7	0.098
High school	2240	65.1	63.6	66.6	
Lower than high school	913	26.5	27.4	25.7	
Father's educational attainment					
College or higher	401	12.5	12.8	12.2	0.512
High school	1857	57.8	57.0	58.7	
Lower than high school	954	29.7	30.2	29.1	

^a^Wave 1 survey: the number of respondents in the 26 Ping-Tung elementary schools in 2008

^b^Wave 2 survey: the number of respondents in the 26 Kaohsiung elementary schools in 2009

Variation in the total sample size across the various characteristics is due to missing data

Table 2 shows the descriptive information of the selected individual characteristics of the third through sixth graders from the 52 elementary schools. In general, the scores for the female students' SHS avoidance confidence were higher than the corresponding scores for the male students' in both surveys. Of all of the students surveyed, the male students had higher current and former smoking rates as compared to the female students in both surveys. Among the males from the two surveys, 4.6% and 3.3% were current smokers, and 12.7% and 10.7% were former smokers. Among the females, 1.5% and 1.7% were current smokers, and 7.8% and 6.5% were former smokers, respectively.

**Table 2:** Descriptive information on selected individual characteristics of the third through sixth graders at the 52 elementary schools in southern Taiwan, 2008 and 2009

Parameter	Wave 1						Wave 2					
	
	Male		Female		*X^2^, t*	*P*	Male		Female		*X^2^, t*	*P*
Children smoking status (n, %)												
Never smokers	976	82.7	997	90.6	34.10	< 0.001	889	86.0	996	91.8	18.57	< 0.001
Former smokers	150	12.7	86	7.8			111	10.7	71	6.5		
Current smokers	54	4.6	17	1.6			34	3.3	18	1.7		
Family smokes (n, %)												
No	395	33.3	352	31.8	2.15	0.543	383	36.4	378	34.2	4.98	0.173
Smoking in front of me												
Never	163	13.8	150	13.6			120	11.4	136	12.3		
Sometimes	346	29.2	354	32.0			273	26.0	328	29.7		
Always	281	23.7	251	22.7			276	26.2	264	23.9		
Household SHS exposure (n, %)												
None	466	40.8	423	39.3	1.32	0.517	429	42.3	454	42.2	0.25	0.883
1-3 days/week	254	22.3	261	24.3			221	21.8	244	22.7		
4+ days/week	421	36.9	391	36.4			363	35.8	378	35.1		
Attitude toward smoking (mean ± SD)	17.06 ± 4.65		16.02 ± 3.92		5.75	< 0.001	16.59 ± 4.65		15.93 ± 4.24		3.42	< 0.001
Knowledge (mean ± SD)	7.42 ± 2.33		7.67 ± 2.02		-2.73	0.006	7.55 ± 2.36		7.71 ± 2.11		-1.65	0.010

The results from both surveys show that 52% and 58% of these children were exposed to household SHS and that 35% of the children were exposed more than 4 days a week. Before implementation of the new Act Amendment, families with school children were found to have a 67-69% smoking rate, and nearly 54% of them had family members that smoked in front of the children. After the new act was implemented, it was found that the smoking rate of families with school children had decreased slightly to 64-66% and 52% of those family reported smoking in front of the children. In the wave 1 survey, the female students were found to have a significantly higher score in regard to their knowledge of tobacco hazards than the male students (7.67 ± 2.02 vs. 7.42 ± 2.33). Nevertheless, the latter reported having a significantly higher score with respect to their attitude toward smoking than the female students (17.06 ± 4.65 vs. 16.02 ± 3.92). In the wave 2 survey, the male students reported that they still had a significantly lower score in the knowledge about tobacco hazards (7.55 ± 2.36 vs. 7.71 ± 2.11) and higher score as to their attitude toward smoking as compared to their female counterparts (16.59 ± 4.65 vs. 15.93 ± 4.24).

### Children's confidence on household SHS exposure avoidance

A multiple regression analysis was then performed with models incorporated with various variables to assess the children's confidence level of household SHS exposure avoidance. The results of the regression analysis illustrated in Tables 3 and 4 are arranged by the model employed, variables considered in model, year of the survey and the gender of participants. After adjusting for potential covariates, results in Table 3 reveals that the characteristics found to be significantly associated with children's avoidance behavior for both genders in wave 1 survey were grade, household SHS exposure, and their attitude toward smoking. Male students from higher grades (β = -0.40 to -0.90), with a household SHS exposure of more than 4 days a week (β = -0.36), and exhibiting a more positive attitude toward smoking (β = -0.06) were found to be have a lower level of SHS avoidance. Male students with a higher knowledge score about tobacco hazards (β = 0.13) were found to be significantly associated with a higher level of household SHS avoidance. Female students from higher grades (β = -0.35 to -1.01), with a household SHS exposure of more than 4 days a week (β = -0.44), and with a more positive attitude toward smoking (β = -0.06) were observed to be significantly associated with a lower level of household SHS avoidance. These variables accounted for 11.9% and 12.3% of the total variance in the level as to avoiding household SHS in male and female students, respectively (Table 4). Adding solely the "attitude toward smoking" factor increased the variance of household SHS avoidance by 2.5% and 1.8% (Model 4) for males and females, respectively. Results of Model 5 suggest that "knowledge" alone accounted for 2.5% of the total variance in the male student's confidence to avoid SHS exposure. The better the knowledge of tobacco hazards was observed to be, the stronger their confidence to avoid SHS was. Among female students, knowledge was not observed to play a significant role in their confidence levels.

**Table 3:** The multiple regression models about confidence level as to the avoidance of household SHS exposure among third through sixth graders in southern Taiwan, arranged according to survey year and gender

Model	Wave 1				Wave 2			
	
	Male		Female		Male		Female	
	**β**	**(95% CI)**	**β**	**(95% CI)**	**β**	**(95% CI)**	**β**	**(95% CI)**
***Model 1***								
Grade								
3	Ref.		Ref.		Ref.		Ref.	
4	-0.40	(-0.71,-0.09)	-0.35	(-0.64,-0.06)	0.17	(-0.16,0.51)	-0.32	(-0.62,-0.03)
5	-0.13	(-0.44, 0.17)	-0.83	(-1.13,-0.53)	-0.09	(-0.43,0.25)	-0.29	(-0.59,-0.00)
6	-0.90	(-1.21,-0.59)	-1.01	(-1.30,-0.72)	-0.27	(-0.60,0.07)	-0.79	(-1.09,-0.50)
School geography								
Urban	Ref.		Ref.		Ref.		Ref.	
Rural	-0.08	(-0.32,0.17)	0.19	(-0.05,0.42)	0.09	(-0.18,0.35)	0.02	(-0.22,0.25)
Mountain	-0.06	(-0.44,0.32)	0.23	(-0.16,0.62)	-0.25	(-0.72,0.23)	-0.04	(-0.48,0.39)
Ethnicity								
Non-aboriginal	Ref.		Ref.		Ref.		Ref.	
Aboriginal	-0.08	(-0.52,0.36)	0.30	(-0.10,0.70)	0.63	(0.24,1.01)	0.32	(-0.04,0.69)
Mother's educational attainment								
College or higher	Ref.		Ref.		Ref.		Ref.	
High school	-0.02	(-0.37,0.34)	-0.27	(-0.62,0.08)	0.10	(-0.24,0.45)	-0.35	(-0.68,-0.02)
Lower than high school	0.24	(-0.22,0.69)	-0.05	(-0.45,0.35)	-0.14	(-0.56,0.29)	0.10	(-0.30,0.49)
Father's educational attainment								
College or higher	Ref.		Ref.		Ref.		Ref.	
High school	0.02	(-0.31,0.36)	0.01	(-0.31,0.32)	-0.14	(-0.46,0.18)	0.11	(-0.18,0.39)
Lower than high school	0.11	(-0.29,0.52)	-0.00	(-0.37,0.37)	-0.12	(-0.52,0.29)	-0.18	(-0.52,-0.16)
Monthly household income								
High	Ref.		Ref.		Ref.		Ref.	
Middle	-0.07	(-0.40,0.26)	0.10	(-0.21,0.40)	-0.24	(-0.57,0.09)	0.00	(-0.30,0.30)
Low	-0.07	(-0.40,0.25)	0.22	(-0.06,0.50)	-0.19	(-0.50,0.12)	0.14	(-0.15,0.43)
***Model 2***								
Children smoking status								
Never	Ref.		Ref.		Ref.		Ref.	
Ever	-0.13	(-0.44,0.19)	-0.29	(-0.66,0.08)	-0.37	(-0.72,-0.01)	-0.11	(-0.52,0.30)
***Model 3***								
Household SHS exposure								
None	Ref.		Ref.		Ref.		Ref.	
1-3 days/week	-0.05	(-0.39,0.28)	-0.15	(-0.47,0.17)	0.08	(-0.27,0.43)	-0.06	(-0.40,0.28)
4+ days/week	-0.36	(-0.68,-0.04)	-0.44	(-0.77,-0.11)	-0.34	(-0.68,0.00)	-0.47	(-0.81,-0.14)
Family smokes								
No	Ref.		Ref.		Ref.		Ref.	
Smoking in front of me								
Never	-0.03	(-0.68,-0.04)	-0.05	(-0.39,0.30)	-0.12	(-0.52,0.28)	-0.05	(-0.41,0.32)
Sometimes	-0.10	(-0.43,0.23)	-0.05	(-0.38,0.28)	0.02	(-0.33,0.36)	-0.28	(-0.62,0.06)
Always	-0.23	(-0.60,0.14)	-0.32	(-0.70,0.07)	-0.27	(-0.64,0.10)	-0.24	(-0.62,0.15)
***Model 4***								
Attitude toward smoking	-0.06	(-0.08,-0.03)	-0.06	(-0.09,-0.03)	-0.05	(-0.07,-0.02)	-0.06	(-0.09,-0.04)
***Model 5***								
Knowledge of tobacco hazards	0.13	(0.09,0.18)	0.05	(-0.01,0.10)	0.14	(0.09,0.19)	0.07	(0.02,0.12)

**Model 1**: This model included socio-demographic factors

**Model 2**: The variables included socio-demographic factors and student smoking behavior

**Model 3**: The variables included socio-demographic factors, student smoking behavior, household SHS exposure, and family smoking

**Model 4**: The variables included socio-demographic factors, student smoking behavior, household SHS exposure, family smoking, and attitude toward smoking

**Model 5**: The variables included socio-demographic factors, student smoking behavior, household SHS exposure, family smoking, attitude toward smoking, and knowledge of tobacco hazards

**Table 4:** The multiple regression models about confidence level as to the avoidance of household SHS exposure among third through sixth graders in southern Taiwan, arranged according to survey year and gender

Order of Entry	Mult. R	R^2 ^(%)	R^2 ^change (%)	Sig. change		
	**Wave 1, Male**					
Model 1	0.20	4.1	4.1	F_change _=	3.90,	*P *< 0.001
Model 2	0.23	5.3	1.2	F_change_=	14.10,	*P *< 0.001
Model 3	0.26	6.9	1.6	F_change _=	3.86,	*P *< 0.001
Model 4	0.31	9.4	2.5	F_change _=	30.16,	*P *< 0.001
Model 5	0.35	11.9	2.5	F_change _=	30.56,	*P *< 0.001
	**Wave 1, Female**					
Model 1	0.26	6.6	6.6	F_change _=	6.13,	*P *< 0.001
Model 2	0.27	7.4	0.8	F_change _=	9.47,	*P *< 0.001
Model 3	0.32	10.3	2.8	F_change _=	6.55,	*P *< 0.001
Model 4	0.35	12.1	1.8	F_change _=	21.29,	*P *< 0.001
Model 5	0.35	12.3	0.3		n.s.	
	**Wave 2, Male**					
Model 1	0.14	2.0	2.0		n.s.	
Model 2	0.20	3.9	1.9	F_change _=	19.09,	*P *< 0.001
Model 3	0.25	6.1	2.3	F_change _=	4.65,	*P *< 0.001
Model 4	0.28	8.1	2.0	F_change _=	20.67,	*P *< 0.001
Model 5	0.33	10.6	2.5	F_change _=	27.40,	*P *< 0.001
	**Wave 2, Female**					
Model 1	0.20	4.1	4.1	F_change _=	3.69,	*P *< 0.001
Model 2	0.22	4.8	0.7	F_change _=	7.58,	*P *< 0.01
Model 3	0.28	8.0	3.2	F_change _=	7.19,	*P *< 0.001
Model 4	0.32	10.4	2.4	F_change _=	27.73,	*P *< 0.001
Model 5	0.33	11.0	0.6	F_change _=	6.63,	*P *< 0.01

**Model 1**: This model included socio-demographic factors

**Model 2**: The variables included socio-demographic factors and student smoking behavior

**Model 3**: The variables included socio-demographic factors, student smoking behavior, household SHS exposure, and family smoking

**Model 4**: The variables included socio-demographic factors, student smoking behavior, household SHS exposure, family smoking, and attitude toward smoking

**Model 5**: The variables included socio-demographic factors, student smoking behavior, household SHS exposure, family smoking, attitude toward smoking, and knowledge of tobacco hazards

In the wave 2 survey, as presented in Table 3, household SHS exposure, the attitude toward smoking, and one's knowledge of tobacco hazards were found to be significantly associated with the confidence level of children's household SHS avoidance for both genders, even after adjusting for other potential variables. Among males, being an ever-cigarette smoker (β = -0.37), in a household with SHS exposure of more than 4 days a week (β = -0.34), and more positive attitude toward smoking (β = -0.05) had a lower confidence level in SHS avoidance. On the other hand, for the aboriginal students (β = 0.63) and for those with a higher knowledge score about tobacco hazards (β = 0.14), a significant association was found with respect to their higher confidence level. Among females, students who were in higher grade (β = -0.29 to -0.79), who had a household SHS exposure of more than four times a week (β = -0.47), and a more positive attitude toward smoking (β = -0.06), were found to be significantly associated with a lower confidence level in regard to SHS exposure avoidance. Knowledge of tobacco hazards was another significant factor in regard to the respective confidence level of household SHS avoidance in female students in the wave 2 survey (β = 0.07). These variables combined attribute 10.6% and 11.0% of the total variance in the confidence level of household SHS avoidance in male and female students, respectively, in the wave 2 survey (Table 4). Adding solely the "attitude toward smoking" factor increased the variance in household SHS avoidance level by 2.0% and 2.4% (Model 4) in males and females, respectively. Results of Model 5 suggest that the degree of their knowledge regarding tobacco hazards accounted for 2.5% and 0.6% of the total variance in their confidence level of SHS exposure avoidance in male and female students, respectively.

## Discussion

### High household SHS exposure

The results indicate that after the new Taiwan Tobacco Hazards Prevention Act was implemented, a significant number of schoolchildren still lived with family member who smokes in front of them and thus were subjected to household SHS exposure. The prevalence of SHS exposure at home is rather high in two waves of surveys (52% and 58%, respectively). In particular, 35% of the schoolchildren were exposed to SHS 4 days (or more) a week. This is much higher than the 25% SHS exposure rate found in the US national study [18]. Since the smoking prevalence rate for Taiwanese women is relatively low as comparing to Taiwanese men, the high smoking prevalence among men can be considered the major risk factor for SHS exposure among the non-smokers [6]. The new 2009 Act protects children from all indoor SHS exposures except exposure that occurs at private residence. This has led to the conclusion that the majority of the SHS exposure in children occurs at the home setting. The adoption and maintenance of a more smoke-free home can be an effective strategy to decrease children's SHS exposure [19].

### Contributing factors associated with household SHS avoidance behavior

This study explored the potential of various factors to predict children's SHS avoidance level. Attitudes toward smoking, as well as the number of days exposing to SHS were found to be significantly associated with the avoidance of SHS in schoolchildren, both before and after the implementation of the new Act. One's attitude toward smoking was the strongest factor that was found to be associated with household SHS avoidance at the confidence level. This finding is consistent with that of previous studies [11,20], which have indicated that attitude works as the better predictor. Attitude comes from the target belief, and each belief and behavior is connected to a specific result and will lead to the implementation of the production [21]. Furthermore, we also found that if the children were exposed to household SHS 4 days a week, it can explain a high degree of the avoidance of children's confidence. This means that, notwithstanding the implementation of the new policy, when the children were exposed to household SHS for a longer amount of days, their confidence level, in regard to being able to avoid SHS in their home, decreased.

Among male students, their tobacco hazard knowledge scores were found to be significantly related to their avoidance behavior. Our study also found that the factor of different genders with respect to their avoidance of SHS also varies. With higher smoking knowledge, male students were found to be more likely to have higher confidence level with respect to being able to avoid SHS. The results of this study are similar to those found by Kurtz et al.[20] Elementary schools with tobacco-related educational activities or counseling had a greater impact on male student's risk behavior [9]. Moreover, as compared to their female counterparts, the mean score of the knowledge of tobacco hazard among male students is lower, further suggesting the likelihood of having a great positive impact if the tobacco awareness and prevention education program is there in a stronger manner for male students toward their avoidance of household SHS.

Our study found that if the male students have been smoking, their confidence level of household SHS exposure avoidance was also found lower. Students who had not been smoking more resolutely believed in their ability to resist smoking than those students who had tried smoking before. In addition, since students who were experienced smokers felt that they could not refuse smoking, it is expected that the latter group would have a lower confidence level, and this also seems to be true for regular smokers [22]. As a result of the new act's implementation, the SHS avoidance behavior with respect to individual smoking behavior for male students has a highly negative correlation.

Schoolchildren from higher grades showed the worst levels of children's confidence that they are currently avoiding SHS exposure. The higher graders might understand that the decision power at home is in their parents' and not their hands, so they tended to accept this fact and their confidence scores gradually declined. This may also be due to the growth of schoolchildren, and since schoolchildren are greatly affected by external temptations [8]. Therefore, such factors will lead to a greater probability of low confidence for students in higher grades.

### Study limitations

There are several research limitations in regard to this study. First of all, since this study used a cross-sectional study design, all of the relations were relevant but the causal relationships cannot be derived. Second, a self-reported questionnaire used to investigate smoking behavior may have the problem of social desirability. However, the responses in this study were taken by secret ballot so as to increase its validity. Finally, the lower reading level in 3^rd^-6^th ^grade students in remote area may have led to the validity issue.

## Conclusions

Our study found and reveals that more than half of the observed elementary schoolchildren were exposed to household SHS, even after new act. The family was observed to be the greatest impact and those that the children spend with most of their time. Therefore, reducing household SHS exposure for schoolchildren has become a quite important goal to the relevant units. The Tobacco Hazards Prevention Act requires the majority of public places to have a clear non-smoking policy, but one's family is in a private place that the act cannot enforce. We conclude that the results provide further evidence that the Tobacco Hazards Prevention Act should perhaps be extended to the family environment in order to protect children from the hazards of household SHS exposure, especially for homes with children below the age of 12. In addition, male's knowledge about smoking hazards was significantly related to their SHS avoidance. The intervention program should enhance schoolchildren do actively avoid exposure to Secondhand Smoke in home settings, and more importantly, provide tobacco hazard knowledge to male students to avoid exposure to household SHS for themselves.

## Competing interests

The authors declare that they have no competing interests.

## Authors' contributions

HLH is the principal investigator of this study. She contributed to the study design, sampling protocol and method development, statistical analysis and interpretation of the results, and is the primary writer of this manuscript. YYY and PLL conceived of the study and revised the manuscript critically for important intellectual content. CHC and CCH conceived of the study and participated in the design of the study, have been involved in drafting and revising the manuscript. TC and CYH revised the manuscript critically for important intellectual content. YYL and CHL assist in statistical analysis, interpretation of data and drafted the statistical analysis of manuscript. FLC conceived of the study, and have made substantial contributions to conception and design, acquisition of data, and revised the manuscript critically for important intellectual content. All authors read and approved the final manuscript.

## Pre-publication history

The pre-publication history for this paper can be accessed here:

http://www.biomedcentral.com/1471-2458/12/40/prepub

## Supplementary Material

Additional file 1 **Table S1**. Definition of independent variables and data scale. Click here for file

Additional file 2 **Table S2**. An overview of the different items in knowledge and attitudinal composite variable. Click here for file
