# Supplementary material for: Household secondhand smoke exposure of elementary schoolchildren in Southern Taiwan and factors associated with their confidence in avoiding exposure: a cross-sectional study
Source: BMC Public Health. 2012 Jan 17;12:40. doi: 10.1186/1471-2458-12-40 (PMC3316143; doi:10.1186/1471-2458-12-40)
Supplement: Additional file 1 — Table S1. Definition of independent variables and data scale. [file 1471-2458-12-40-S1.DOC]

**Table S-1** Definition of independent variables and data scale

| **Variables** | **Definition** | **Data Scale** |
| --- | --- | --- |
| Socio-demographic factors |  |  |
| Age | Year of birth | Continuous |
| Grade | Elementary grade (3-6) | Ordinal |
| Gender | Boy/ Girl | Nominal |
| Ethnicity | Aboriginal/ Non-aboriginal | Nominal |
| School geography | Geographical distribution (Urban area/ Rural area/ Mountain area) | Nominal |
| Monthly household income* | Average monthly household income (<$NT19, 999/ $NT 20,000-39,999/ $NT 40,000-59,999/ $NT 60,000-79,999/ $NT 80,000-99,999/ $NT 100,000-119,999/ $NT 120,000-139,999/ $NT 140,000-159,999/ >$NT 160,000) | Ordinal |
| Parental educational attainment | “*What is subject’s father the highest education attainment?*” and “*What is subject’s mother the highest education attainment?*”  Elementary school (≤ 7 years)/ Junior high school (7-9 years)/ High school (10-12 years)/ Occupational school/ Junior college/ College/ Graduate school and above. | Ordinal |
| Secondhand Smoke factor |  |  |
| Household SHS exposure | *Over the past week, how many days did people smoke in front of you while you were at home?* (‘None’/‘1-3 days’/‘4-6 days’/‘Everyday’) | Ordinal |
| Children’s smoking status | *Have you ever smoked cigarettes (even just one puff)?*(‘I do not smoke’/‘less than one cigarette’/‘less than 10 cigarettes’/‘more than 10 cigarettes’)  *Have you smoked cigarettes over the past month?* (‘No’/’Occasionally’/’ Everyday’) | Ordinal |
| Family smokes | *Is there anyone living in the same household with you that smokes?* (‘Yes’/’No’)  *How often do they smoke in front of you?* (‘Always’/’Sometimes’/’Never’). | Ordinal |
| Psychological factors |  |  |
| Knowledge of tobacco hazards | Ten statements are illustrated in Table S-2 | Continuous |
| Attitude toward smoking | Twelve statements are illustrated in Table S-2 | Continuous |
