# Supplementary material for: Household secondhand smoke exposure of elementary schoolchildren in Southern Taiwan and factors associated with their confidence in avoiding exposure: a cross-sectional study
Source: BMC Public Health. 2012 Jan 17;12:40. doi: 10.1186/1471-2458-12-40 (PMC3316143; doi:10.1186/1471-2458-12-40)
Supplement: Additional file 2 — Table S2. An overview of the different items in knowledge and attitudinal composite variable. [file 1471-2458-12-40-S2.DOC]

| **Table S-2** An overview of the different items in knowledge and attitudinal composite variable |
| --- |
| **Composite variable: Knowledge of Tobacco Hazards - KR-20 coefficient: 0.76** |
| As long as we do not inhale secondhand smoke, smoking is not dangerous  The younger one starts smoking, the higher the risk is for cancer  As long as I do not smoke, I will not be the victim of smoking, even if I am with people who smoke  It is possible to be addicted to smoking  If smoking in an outdoor area with fresh air, one does not have to worry about health damage due to smoking  Smoking helps prolong the lifespan (one may live longer)  Smoking contributes to a high incidence of blood vessel occlusion (the blood vessel is blocked and the blood cannot flow smoothly)  Smoking makes people suffer from bronchitis (a kind of respiratory diseases)  Smoking pollutes the air  Smoking makes one’s teeth turn yellow |
| **Composite variable: Attitude toward Smoking- Cronbach’s alpha: 0.73** |
| Smoking makes people happy  Smoking costs a lot of money  Smoking helps relieve loneliness  Smoking means being a grown-up  Smoking helps exert one’s imagination  Smoking makes people look cooler  Smoking is interesting  Everyone likes to get along with people who smoke  Smoking is a brave behavior  Parents should forbid children to smoke  No one is allowed to smoke in schools  Parents can smoke in front of children |
